# Frequency of GP communication addressing the patient's resources and coping strategies in medical interviews: a video-based observational study

**DOI:** 10.1186/1471-2296-10-49

**Published:** 2009-07-01

**Authors:** Trond A Mjaaland, Arnstein Finset

**Affiliations:** 1 Department of Behavioural Science in Medicine, Institute of Basic Medical Sciences, Faculty of Medicine, University of Oslo, Oslo, Norway

## Abstract

**Background:**

There is increasing focus on patient-centred communicative approaches in medical consultations, but few studies have shown the extent to which patients' positive coping strategies and psychological assets are addressed by general practitioners (GPs) on a regular day at the office. This study measures the frequency of GPs' use of questions and comments addressing their patients' coping strategies or resources.

**Methods:**

Twenty-four GPs were video-recorded in 145 consultations. The consultations were coded using a modified version of the Roter Interaction Analysis System. In this study, we also developed four additional coding categories based on cognitive therapy and solution-focused therapy: attribution, resources, coping, and solution-focused techniques.

The reliability between coders was established, a factor analysis was applied to test the relationship between the communication categories, and a tentative validating exercise was performed by reversed coding.

**Results:**

Cohen's kappa was 0.52 between coders. Only 2% of the utterances could be categorized as resource or coping oriented. Six GPs contributed 59% of these utterances. The factor analysis identified two factors, one task oriented and one patient oriented.

**Conclusion:**

The frequency of communication about coping and resources was very low. Communication skills training for GPs in this field is required. Further validating studies of this kind of measurement tool are warranted.

## Background

Over the last few years, we have seen an increasing focus on patient centeredness and shared decision making in primary care [1]. The research literature has reported that these developments have a number of advantages in patient care, including greater satisfaction with the decision process, more realistic expectations, increased correlation between patient values and decisions, and more active participation in the decision-making process by a patient [2]. A common feature in these trends is the greater emphasis on patient involvement, in terms of both eliciting a patient's perspective, the active transfer of information, and greater emphasis on shared decision making.

Patient involvement is further refined in the field of psychotherapy. Several current psychotherapeutic methods aim to help patients overcome and resolve problems by using a patient's own resources in different ways, e.g., solution-focused therapy [3].

Our research group is involved in the development and assessment of training general practitioners (GPs) in communication skills. We emphasize skills that enhance patients' awareness of and focus on their own resources, in addition to the general biomedical focus of the consultation. When we started to develop a communication skills training programme, we assumed that it is relatively rare in general practice for the physician's communication behaviour to be systematically directed towards a patient's resources and coping strategies. However, this assumption was based on anecdotal evidence. Although several studies have documented the amount of psychosocial content of consultations, they have not specified how much of the psychosocial content is focused on patients' personal resources and coping behaviour rather than on their psychological distress and other problems [4,5]. We consider this significant because the GP must know a patient's coping skills to be able to evaluate them and act accordingly. According to Di Caccavo et al., patients who are prescribed drugs are asked less frequently about psychological coping strategies than are patients given non-drug treatments [6]. Coulter and Elwyn [7] even argue that strengthening a patient's coping skills could help to reduce the workload of the GP. Therefore, we decided to analyse the frequencies of various specific categories of psychosocial communication in general practice. Such data should provide both specific insight into the physicians' discussion of patients' personal resources and coping behaviour, and a baseline to which we can later refer in communication skills research.

To the best of our knowledge, none of the existing methods of interaction analysis in medical interviews specifically assesses this sort of communication in the desired detail. Consequently, we developed an approach to interaction analysis in terms of a one-channel system, and coded all GP communication to quantify the number of times a GP states a question or a comment or gives an explanation, according to specific content categories.

We took as the point of departure the system most frequently applied to measure the doctor-patient interaction, the Roter's Interaction Analysis System (RIAS) [8]. Because none of the RIAS categories specifically emphasizes patient resources, we added four new categories to assess the relevant aspects of the psychosocial content: (i) attribution (A), (ii) an explicit focus on personal resources (R), (iii) coping (C), and (iv) the technically oriented solution-focused and cognitive behavioural therapy (CBT) techniques of scaling, exceptions, summing up, and homework (S). We used the acronym ARCS to denote these four categories. Our research questions were:

1. Can resource- and coping-oriented communication by a physician (ARCS), as defined by the interaction analysis system, be reliably and validly measured based on the video analysis of medical interviews?

2. How frequently do units of resource- and coping-oriented communication (ARCS) by a physician occur in consultations in general practice?

3. How is resource- and coping-oriented communication (ARCS) by a physician related to the more general categories of doctor communication, as measured with RIAS?

## Methods

### Subjects

Eighty GPs in the municipality of Bærum, Norway, were invited to participate in the study, without having to pay for the training. After a baseline assessment of the physicians' communication behaviour in the consultations, the participants would take part in a communication skills training program, followed by a second assessment of their communication. A thorough description has been submitted for publication elsewhere. The training programme was approved by the Norwegian Medical Association as part of the training that leads to the accreditation or the continued accreditation of specialists in general medicine. Hence, the sample is a convenience sample based on a window of opportunity in the community and on decisions made by the local health authorities. We wanted as many GPs and patients as possible to participate in the study.

Twenty-seven GPs were enrolled in the study. After enrolment, three GPs withdrew from the study, leaving 24 GPs enrolled, nine women and 15 men. Each GP chose a day for the video recordings within a one-month time period. Each GP sent out invitations, including informed consent forms, to all the patients who had appointments for consultations on that day, one week in advance. The excluded patients were more than 80 years old or less than 18 years old, and those patients who had booked immediate-assistance consultations on the same day. This resulted in a total number of 145 patients in the study, with a mean number of six patients per GP.

### Interaction analysis

The two most common traditions in measuring physician-patient communication behaviour are utterance-by-utterance coding and rating-scale techniques [9]. We decided on detailed utterance-by-utterance measurements of the GP communications because we wanted to focus on the frequency of each specific behavioural category and the possible patterns within and between them.

The overall physician communication behaviour in all 145 consultations was coded according to a modified version of RIAS. We collapsed several categories of the RIAS system, making the categories broader. These were collected into 10 categories, which we refer to as "general RIAS communication codes". Collapsing categories is the rule rather than the exception in studies like this [4]. Most often, the collapsing of categories is applied in analyses based on observations using all categories. The effects on the reliability of the collapsed categories during coding are not known, but there is no reason to believe they have any negative effect. The categories and their corresponding RIAS categories are outlined in Table 1.

**Table 1:** General RIAS communication codes.

Name	Description	Example	Corresponding RIAS codes
Social talk	All aspects of social conversation, personal remarks, laughter, and compliments, not related to the patient's health status.	So, you managed to get here in time despite the heavy snow this morning – exhausting, eh?	Personal remarks, social conversation Laughs, tells jokes

Biomedical questions	All questions regarding symptoms, illness, or biomedical treatment.	How is your lower back pain?	Asks questions (open or closed ended) - medical condition - therapeutic regimen

Biomedical information	Facts and figures, advice, opinions and suggestions regarding the patient's biomedical health status.	You need to take these pills to relax your tense muscles	Gives information/counsels - medical condition - therapeutic regimen

Questions about lifestyle and psychosocial issues	All questions regarding psychological, lifestyle, social or other non-biomedical issues.	Any progress on your weight-loss programme so far?	Asks questions (open or closed ended) - lifestyle - psychosocial - other

Information about lifestyle and psychosocial issues	Facts and figures, advice, opinions and suggestions regarding psychological, lifestyle, social, or other non-biomedical issues.	You see, the strain on your lower back gets more intense the more you gain weight.	Gives information/counsels - lifestyle - psychosocial - other

Gives orientation	All sorts of instructions or direction of patient actions during the examination.	Please take off your shirt so I can take a look.	Gives orientation Transition words

Facilitation	Process-oriented utterances, more than two words, or comments or questions to facilitate the interview or check on understanding.	All right, I see...	Shows agreement Backchannel responses Paraphrases, checks for understanding Bid for repetition Asks for understanding Asks for opinion

Empathy	An emotionally laden supportive utterance or comment to confirm the patient's speech.	Oh yeah, that must be really painful	Empathy Reassurance, optimism Legitimizes Partnership Shows approval

Shows disapproval	Comments of criticism, anger or hostility.	You must stop smoking!	Shows disapproval Shows criticism

Residual, Unintelligible	Phone calls during the consultation or other irrelevant utterances. Coder not able to understand the GP's speech.		

Pooled codes applied in the study and corresponding RIAS codes.

### Four additional codes

In operationalizing the physician content with reference to a patient's personal resource issues for the coding scheme, we chose a series of content categories that we considered to correspond a priori to specific aspects of the content in the area of personal resources, partly on the basis of the principles of solution-focused therapy [10] and CBT [11]. We then constructed a series of verbal expressions in each content category as examples for coding. Some of these expressions were taken from the relevant research literature, whereas others were defined by the research team. During coding, new examples with verbatim expressions taken from the consultations were added as examples. The new ARCS categories are specified in Table 2 and examples of the content of each category are given.

**Table 2:** ARCS communication codes.

Name	Description	Examples
Resources	When the GP comments on what he/she might consider a positive asset or situational aspect of the patient's health status.	Well, it is nice to have your grandchildren around! That must be nice, don't you agree?

Coping	Questions or comments regarding how the patient has managed to cope, not to give up, or not to break down completely.	How do you manage all this pain? What kind of resources do you have that help you withstand the pain?

Attribution	Questions or comments discussing what the patient thinks about their own situation to uncover other, more helpful thoughts and interpretations.	Yeah, that's interesting. From where did you get that idea? Are there other possible ways of explaining your experience?

Solution-focused techniques	Intervention to place the patient's health status on a scale from zero to 10, where zero is as troublesome as it has ever been, and 10 is as good as it gets.	On a scale from zero to 10, where zero is as troublesome as it has ever been, and 10 is as good as it gets, where would you say you are, right now?
	Questions and comments to explore exceptions to the pressure of the symptom, where the symptoms are less or even gone.	Are there any circumstances under which the lower-back pain is less or even gone?
	The GP addresses issues from the consultation, by using the patient's own phrases, to summarize, focus the consultation, or decide the next step	So, let's see... you have managed to find a way to decrease your lower back pains and you have even discovered some distracting behaviour so you sort of forget the pain. Is that right?
	The GP alone or in discussion with the patient defines homework prior to the next session.	Are there any issues you would like to explore a bit more until we meet next Friday?

### Units of analyses

We analysed GP speech in terms of utterances and turns. An utterance was defined as the smallest meaningful verbal expression. The next level of analysis in the conversation is the "turn". A turn is when the GP talks, "has the floor", and drives the communication. A turn may consist of only one utterance, but it normally includes several utterances.

Our default unit of coding was the turn. If the same code could account for the whole turn, the turn was coded as one unit. If the coding category content changed within the turn, more than one code was applied to that turn.

Minimal expressions from the doctor in terms of back-channelling continuers ("ok", "yes", "hmm", etc.) during a patient's turn were not coded [12].

### Reliability of the coding scheme

Kappa was used to test the reliability of the distinction between the ARCS codes and the general RIAS codes. A team of five coders met to discuss the content of and the inclusion and exclusion criteria for each of the categories. The team then commenced a series of meetings in which individually coded consultations were recoded during a discussion of the coding practice (consensus coding). Detailed definitions of the categories were further developed through discussion. At every meeting, Cohen's kappa values [13] were measured for the individually coded consultations until satisfactory reliability was established between the coders. The group required five consensus codings to establish satisfactory reliability. At that point, one researcher (T.M.) stepped out of the group. His coding was from then on considered the reference coding for the team of coders. Eleven random consultations were then coded individually and the kappa values were established between the reference coder and the other coders. At this point, kappa values were satisfactory between the general RIAS communication and the ARCS communication. However, the kappa values of three of the ARCS categories were not optimal, so a new consensus coding was established. At this point, the consensus coding stopped and individual coding continued. After approximately 10% of the consultations had been coded, the kappa estimates were made to check that the reliability still was satisfactory. Before the statistics were computed, we removed the turns from the data when there was any inconsistency in the observation of turn taking, e.g., where one coder coded a turn, but the other coder did not. This was done, as described above, because the unit of analysis was the content of the turn rather than the turn itself.

All consultations that had been part of the consensus coding were included in the pool of consultations and recoded approximately five months after the first review. This was done because the base rate of the resources communication in the material was low and also because there was no other material to use for the coding exercises.

### Validity of the coding scheme

An important aspect of validity in the measurement of communication is the extent to which the measured communication taps the meaning of the theoretical definition. To understand more about this validity issue, we performed a reversed coding exercise. This exercise was used to demonstrate that there was no conceptual contamination between the ARCS categories and the general RIAS communication categories. Five researchers, experienced in the communication skills training of medical students but without any knowledge of the assessment concept, were given an overview of the names of all the communication categories we had originally used, for all 16 categories (collapsed RIAS and ARCS categories), together with two cards from each category: one definition and one criterion card. The cards were presented in random order. The researchers were then asked to categorize the cards under the correct category label, together with the correct behaviour criterion.

During coding, the category "residual, unintelligible" was added. In the statistical analysis, the four sub-categories comprising the C-code were merged into one code because they were used infrequently in the consultations. This resulted in the 14 categories presented in Tables 1 and 2.

### Statistics

To test the communication patterns in the use of the ARCS categories and the general RIAS codes, we conducted a factor analysis (principal components analysis, with varimax rotation) of all 145 consultations and all communication categories, except for the "shows disapproval" and "residual, unintelligible" categories. These were excluded because of the low sample number, and the lack of relevance for the analysis. The numerical requirements were met with an n value of more than 10 times the number of items in the analysis.

Observer XT, version 6.4.1, was used for coding [14]. Statistical calculations were performed using the Statistical Package for the Social Sciences 14.0 [15].

### Ethical approval

The study was approved by the regional committee for research ethics and the Norwegian Social Science Data Services.

## Results

### Results of reliability measurements

Ten percent of all consultations were coded in the reliability test. There was agreement across coders that 1778 utterances should be coded with non-ARCS codes, whereas 25 utterances should be coded in the ARCS categories. There was disagreement on 45 utterances, coded as ARCS utterances by one of the coders. When the agreed codes (1778 and 25) were added and divided by the total number of coded utterances (1848), there was 98% agreement among the coders. The reference coder applied a consistently more conservative coding. Cohen's kappa was 0.52.

### Validity

With two exceptions, all cards were classified correctly. One coder mixed the definitions of two general RIAS codes; another coder similarly mixed two of the ARCS codes. Thus, there was no misclassification across the general RIAS codes or the ARCS codes.

### Results of the communication assessment

A total of 8741 utterances were coded for the 25 GPs during 145 consultations. The frequencies of each of the measured communication categories are presented in Table 3. One hundred and eighty-five utterances (2.1%) were coded in the ARCS categories.

**Table 3:** All communication categories.

	N	Minimum	Maximum	Mean	Standard Deviation	Sum
All turns	145	7	198	60,2	27,3	8741

All general RIAS communication codes	145	6	194	59,0	26,4	8556

All ARCS communication codes	145	0	33	1,2	3,4	185

Social talk	145	0	31	3,9	5,1	577

Biomedical questions	145	0	49	10,8	7,5	1578

Biomedical information	145	0	62	13,9	9,9	2022

Questions about lifestyle and psychosocial issues	145	0	62	7,9	8,4	1153

Information about lifestyle and psychosocial issues	145	0	41	6,0	7,2	871

Gives orientation	145	0	27	7,5	5,5	1101

Facilitation	145	0	35	6,3	5,6	917

Empathy	145	0	14	2,2	2,7	326

Shows disapproval	145	0	2	0,1	0,2	11

Residual, unintelligible	145	0	18	3,2	3,1	475

Presented in mean values per consultation across the 145 consultations.

### Factor structure

The scree plot indicates a two-factor solution. The first factor, which accounted for 24.6% of the variance, consisted of all the ARCS codes, questions, and information on psychosocial and lifestyle issues and empathy. The second factor, which accounted for 18.7% of the variance, consisted of the facilitation category and task-oriented RIAS communication codes (biomedical questions and information), as well as the category "gives orientation". Social talk did not load either factor. The results are presented in Table 4.

**Table 4:** Rotated component matrix.

	Component	
	
	1	2
Resources	,83	-,09

Coping	,78	-,12

Information about lifestyle and psychosocial issues	,76	-,12

Questions about lifestyle and psychosocial issues	,61	,11

Solution-focused techniques	,45	-,02

Empathy	,41	,36

Attribution	,31	,08

Facilitation	,13	,79

Biomedical information	-,15	,77

Biomedical question	-,16	,68

Gives orientation	,01	,61

Social talk	,05	,25

Extraction method: principal components analysis. Rotation method: varimax with Kaiser normalization.

The use of ARCS communication categories was not evenly distributed among the GPs. There was a borderline significant correlation (*P *< 0.051) between the use of the ARCS codes and the total number of coded behaviours per consultation.

When we aggregated the scores to control for the length of the consultation in terms of the number of coded utterances, six GPs contributed 59% of the ARCS codes in the material. No other distinguishing factor in the available data was identified among those six GPs.

## Discussion

This study indicates that the communication by the physician that emphasizes the personal resources and coping strategies of a patient occurs relatively seldom in general practice, representing 2.1% of the GP's utterances in an average consultation.

### Use of existing vs the development of tailored measurement tools

There are many communication measurement tools available, many with established measures of reliability and validity [16]. A recently developed instrument, the LIV-MAAS [17,18], includes items that address to some extent patients' personal resources and coping mechanisms. However, the LIV-MAAS is a rating scale and as such, is less suitable for the analysis of the frequency of the target utterances in this study. Therefore, none of these tools measures the communication content in the way we required for our research purpose.

### Is this reliably and validly measured?

A kappa value of 0.52 between the proper coders and the reference coder is considered fair, given the review by Bakeman [19]. However, how a kappa calculation is interpreted must be seen in the context of what is measured and the actual figures of the calculation. There was of course a borderline issue among the coders regarding what was included as ARCS utterances, because the variation in the expression of resources and coping utterances is hard to define or categorize precisely. More work is required to define and categorize comments relating to the resources and coping strategies of a patient, so that the ARCS codes can be applied with greater confidence in future work. First and foremost, there was agreement regarding the fact that ARCS communication was scarce in the material, as indicated by the percentage of agreement in the kappa calculation, it is also important to keep in mind the large variety of topics discussed in GP and the impact this has on the categorization of the GP's utterances. The reliability would probably be higher with one category of consultations.

It is interesting that so little of the psychosocially oriented content was related to a patient's personal resources or coping behaviour. The data indicate that the agreement was least between the ARCS codes and the GP's questions and information on lifestyle and psychosocial issues. If the special ARCS codes had not been applied, most of the utterances coded in the ARCS categories would have been coded as questions and information on lifestyle and psychosocial issues. These two coding categories represented 23.2% of all utterances.

The tentative validation exercise for the ARCS categories in this study is only a validation to the extent that it describes the utterances as observed communication intended to help a patient focus on their resources and coping strategies. Whether this is actually the case or not is another issue. To answer this, we would have had to have asked the patients, which was beyond the scope of this study. However, the reversed coding exercise clearly indicated that the meanings of the descriptive words and phrases used in the coding scheme were correctly understood by the professionals in the field.

### Importance and relevance of 2.1%

The ARCS communication was used in 2.1% of the consultations in this study. This frequency warrants two remarks. A measure of frequency does not say anything about the importance or relevance of the communication. In terms of a rare communicative phenomenon, it is often argued by others that, for example, the low frequency at which patients express their emotional cues and concerns is hugely important, even though such expression is a rare phenomenon [20]. In our opinion, information about a patient's coping mechanisms and their use of personal resources is very important because it taps the uniqueness of individual patients, rather than the commonality a patient shares with other patient groups, which is important in the search for a diagnosis. Hence, this uniqueness is a rich source of information upon which to base individual recognition and acknowledgement, and in the search for appropriate solutions to the health needs of individual patients [21].

In terms of a biomedical regime, 2.1% might seem appropriate. However, studies have shown that as many as 50% of patients visiting a GP have no biomedically explicable symptoms [22]. From this perspective, we found that the focus on a patient's resources and coping strategies seemed very low. Coping behaviour and resources are also highly relevant in many biomedical conditions, such as distraction, which is a positive coping mechanism in patients with chronic pain [23].

### ARCS categories and their relationship to general communication

The discussion by physicians on personal resources and coping strategies is part of a general pattern in consultations. Factor analysis indicated that in consultations in which the ARCS categories are used, the GPs tended to ask more questions and supply more information about psychosocial and lifestyle issues, and to make empathic statements. We labelled this general communication pattern "patient-centred communication". How can we understand this?

ARCS communication is not a "decision aid" per se [2], because it is not intended as a tool for decision making, but as a way for a patient to understand their own choices, options, and resource utilization. The ARCS communication categories do not represent "shared decision making" as such [24], because there are not necessarily any joint GP-patient decisions to be made about treatment, except perhaps for the very decision to focus on a patient's personal resources.

The ARCS communication categories may be considered patient-centred [1], but the emphasis on personal resources is not always included in the definition of patient centredness, nor does it share an equal status with illness in terms of "patient centredness" in the consultation [25]. We argue instead that the ARCS communication categories add a new dimension to the concept of patient centredness. As the data indicate, the non-biomedical communication and the ARCS communication constitute a factor in the material.

### Limitations

This study must be considered a pilot study, undertaken to extend our knowledge of specific communication patterns directed towards a patient's resources and coping strategies in general practice. In addition to the abovementioned arguments, we must keep in mind that this study was conducted in a specific suburban area. The participating GPs were not randomly selected, but were invited based on their geographic location. A study from the UK indicated that GPs who agree to be video recorded might differ from non-participating GPs [26]. Another study argued that video-recordings might affect the sampling of patients [27]. These factors alone might be important in dictating the nature of the consultations and the kinds of communication used by the GP. Common sense suggests that most of the consultations are with GPs who feel safe enough to be video recorded and analysed, meeting patients with good relationships with them, and discussing topics that are not emotionally loaded. Conclusions must be drawn accordingly.

Regardless of its frequency, the presence of ARCS communication does not give any information about the quality of care, because that would require a qualitative analysis of the purposefulness or other effect measures. The ARCS communication appears in a particular context, that is, together with more patient-oriented non-biomedical communication, as in factor 1 of the factor analysis. Because of the low base rate of ARCS utterances and the very large standard deviations of these utterances, we must be careful in drawing conclusions based on this factor analysis.

## Conclusion

In this study, discussion by the physician that emphasized the personal resources and coping strategies of a patient was a rare phenomenon. Based on our data, we draw a two-fold conclusion: more communication skills training is needed by GPs to provide them with communication tools designed to address patients' resources and coping abilities, and further studies are required to validate the measurement of these communication categories.

## List of abbreviations

GP: general practitioner; RIAS: Roter Interaction Analysis System; ARCS: attribution, resources, coping, and solution-focused techniques; UK: United Kingdom; CBT: cognitive behavioural therapy.

## Competing interests

The authors declare that they have no competing interests.

## Authors' contributions

TM and AF contributed equally to the statistical analysis and the writing of the article. Both authors read and approved the final manuscript.

## Authors' information

Cand. Psychol. TM is a doctoral fellow and Dr. Philos. AF is a professor in medical psychology.

## Pre-publication history

The pre-publication history for this paper can be accessed here:



## References

[B1] Stewart M (2001). Towards a global definition of patient centred care. BMJ.

[B2] Thistlethwaite J, Evans R, Tie RN, Heal C (2006). Shared decision making and decision – a literature review. Aust Fam Physician.

[B3] Greenberg G, Ganshorn K, Danilkewich A (2001). Solution-focused therapy. Counseling model for busy family physicians. Can Fam Physician.

[B4] Bensing JM, Roter DL, Hulsman RL (2003). Communication patterns of primary care physicians in the United States and the Netherlands. J Gen Intern Med.

[B5] Roter DL, Stewart M, Putnam SM, Lipkin M, Stiles W, Inui TS (1997). Communication patterns of primary care physicians. JAMA.

[B6] Di Caccavo A, Ley A, Reid F (2000). What do general practitioners discuss with their patients? Exploring the relationship between content of medical consultations and treatment decisions. J Health Psychol.

[B7] Coulter A, Elwyn G (2002). What do patients want from high-quality general practice and how do we involve them in improvement?. British Journal of General Practice.

[B8] Roter DL (2008). HTTP://www.rias.org/manual.

[B9] Carroll GJ (1995). The Medical Interview.

[B10] De Jong P, Berg IK (2007). Interviewing for Solutions.

[B11] Keith S, Dobson KS (2003). Handbook of cognitive-Behavioral Therapies.

[B12] Svennevig J (2001). Abduction as a methodological approach to the study of spoken interaction. Norskrift.

[B13] Shoukri MM (2004). Measures of Interobserver Agreement.

[B14] (2007). The Observer. XT[6.4.1].

[B15] (2008). SPSS. [14].

[B16] Boon H, Stewart M (1998). Patient-physician communication assessment instruments: 1986 to 1996 in review. Patient Educ Couns.

[B17] Enzer I, Robinson J, Pearson M, Barton S, Walley T (2003). A reliability study of an instrument for measuring general practitioner consultation skills: the LIV-MAAS scale.[see comment]. Int J Quality Health Care.

[B18] Robinson J, Walley T, Pearson M, Taylor D, Barton S (2002). Measuring consultation skills in primary care in England: evaluation and development of content of the MAAS scale.[see comment] [erratum appears in *Br J Gen Pract *2002;52(485):1026.]. Br J Gen Pract.

[B19] Bakeman R, Harry T Reis, Charles M Judd (2000). Behavioural observation and coding. Handbook of Research Methods in Social and Personality Psychology.

[B20] Zimmermann C, Del Piccolo L, Finset A (2007). Cues and concerns by patients in medical consultations: A literature review. Psychol Bull.

[B21] Smedslund J (2008). Har du sluttet å slå din kone? Ja/Nei. Kan psykologisk prakis være evidensbasert? Ja/Nei. Tidsskrift for den Norske Psykologforening.

[B22] Ring A, Dowrick CF, Humphris GM, Davies J, Salmon P (2005). The somatising effect of clinical consultation: what patients and doctors say and do not say when patients present medically unexplained physical symptoms. Soc Sci Med.

[B23] Affleck G, Urrows S, Tennen H, Higgins P (1992). Daily coping with pain from rheumatoid arthritis: patterns and correlates. Pain.

[B24] Charles C, Gafni A, Whelan T (1997). Shared decision-making in the medical encounter: what does it mean? (or it takes at least two to tango). Soc Sci Med.

[B25] Mead N, Bower P (2002). Patient-centred consultations and outcomes in primary care: a review of the literature. Patient Educ Couns.

[B26] Coleman T (1996). Sampling for qualitative research using quantitative methods. 2. Characteristics of GPs who agree to video-taping of consultations. Fam Pract.

[B27] Coleman T, Manku-Scott T (1998). Comparison of video-recorded consultations with those in which patients' consent is withheld. Br J Gen Pract.

